# Association Between Antidepressant Efficacy and Interactions of Three Core Depression-Related Brain Networks in Major Depressive Disorder

**DOI:** 10.3389/fpsyt.2022.862507

**Published:** 2022-03-08

**Authors:** Qiang Wang, Shui Tian, Peng Zhao, Qiuyun Cao, Qing Lu, Zhijian Yao

**Affiliations:** ^1^Department of Medical Psychology, Nanjing Drum Tower Hospital, The Affiliated Hospital of Nanjing University Medical School, Nanjing, China; ^2^Department of Radiology, The First Affiliated Hospital of Nanjing Medical University, Nanjing, China; ^3^School of Biological Sciences & Medical Engineering, Southeast University, Nanjing, China; ^4^Department of Psychiatry, The Affiliated Brain Hospital of Nanjing Medical University, Nanjing, China

**Keywords:** default mode network, central executive network, salience network, magnetoencephalography, antidepressant efficacy

## Abstract

**Background:**

The central executive network (CEN), salience network (SN), and default mode network (DMN) are the three most studied depression-related brain networks. Many studies have shown that they are related to depression symptoms and treatment effects. However, few studies have related these three networks and their activity frequency bands to depressive symptoms and treatment efficacy.

**Methods:**

Sixty-six medication-free patients with major depressive disorder (MDD) were enrolled. Magnetoencephalography (MEG) was administered at baseline to calculate imaging indicators such as the power and functional connectivity (FC) of each brain network. The Hamilton Rating Score for Depression (HRSD-17) was assessed at baseline and weekly for 4 weeks. Pearson correlation and receiver operating characteristic curves (ROC) analyses were used to explore the relationship between brain imaging indicators and antidepressant efficacy.

**Results:**

The difference between therapeutically effective and ineffective groups was mainly manifested in the beta power of the SN. The FC of beta waves between the three networks was related to antidepressant efficacy, with ROC analysis results of AUC = 0.794, *P* = 0.004, sensitivity = 76.7%, and specificity = 81.8%.

**Limitations:**

The sample size was small and a healthy control group was not available.

**Conclusions:**

The interaction between the three networks is related to antidepressant efficacy and the relief of depressive symptoms.

## Introduction

The prevalence of depression is increasing year by year, and society is paying increasing attention to this important issue. Medications for depression usually take 2–4 weeks to work (1), which can be a long time for patients and their families in need of relief. Waiting without knowing the results can cause patients to lose confidence and cause families to become anxious and distrust the prescribing doctor, leading to poor treatment outcomes. The ability to predict the efficacy of antidepressants at the time of prescription could alleviate this embarrassing situation, increase compliance, and make treatment more targeted.

The imaging of brain networks is an important method for research on efficacy of depression treatment. Among them, three core brain networks are the most studied, namely the default mode network (DMN), central executive network (CEN), and salience network (SN) at baseline (2). Each of these three networks plays a role in the onset, symptoms, and antidepressant efficacy of depression. The DMN comprises cornerstone areas of the brain that are mainly activated at rest. It is related to self-reference, self-reflection, and emotional management (3), and plays an important role in depression. The functional connectivity (FC) of the core brain area in the DMN of depression patients is higher than that of healthy controls (4). A meta-analysis (5) showed that hyperconnectivity within the DMN predicts the response to antidepressant treatment. The CEN, which is composed of the dorsolateral prefrontal cortex (dlPFC), anterior cingulate cortex, and parts of the parietal lobe (6), is also a network closely related to the pathological mechanism of major depressive disorder (MDD). Alexopoulos et al. (7) found that lower CEN connectivity predicted poorer antidepressant efficacy. The SN network can allocate attention to important external stimuli (8), and the dysfunction of this network may be the cause of negative attentional bias in depressed patients (9). The SN network participates in switching between the DMN and CEN (10). SN activity is associated with treatment refractory and may be an imaging marker that can predict recurrence of MDD (11).

Current neurophysiological research tends to study the brain as a complex whole, which contains many brain networks that cooperate and interact with each other, while disturbances in these connections can lead to psychiatric symptoms.

One study found that the connection between the post DMN and right CEN stayed in a more functional and less variable state in patients with MDD than in healthy ones (12). In the Han Chinese ethnic group, Zhi et al. (13) found dynamic functional network connectivity of depressed patients was more in a weekly connected state, mainly involved regions related to the CEN and DMN, which was associated with depression severity and cognitive function. The above research shows that analyzing the interaction between the three core brain networks involved in depression can provide novel insights into its pathophysiological mechanisms. Furthermore, it can provide a theoretical basis for predicting the efficacy of antidepressant treatment (14).

Beta and alpha neural oscillations are involved in the three core brain network activities of depression (15, 16). In the resting state, beta waves occur in the main part of the posterior brain area and are one of the characteristic manifestations of depression. They participate in emotional response (17) and bottom-up emotional cognitive regulation (18), and may be related to the patient's alertness and anxiety (19). It has also been clinically observed that beta activity is positively correlated with the number of depression episodes and can be used to distinguish depressed patients from healthy controls (20). High beta power at baseline can predict future symptomatic relief in depressed patients (21). Alpha oscillations usually reflect the inhibition of brain activity (22) and are closely related to attention regulation, working memory, and executive function (23, 24). Previous studies have shown that excessive alpha wave activity often indicates better antidepressant efficacy (25), while successful antidepressant treatment is accompanied by a decrease in alpha energy (26).

This study observed brain activity at baseline to explore the relationship between antidepressant efficacy and the interaction between the three core brain networks. We aimed to find predictors of antidepressant treatment outcomes and improve the quality of clinical diagnosis and treatment.

## Experimental Procedures

### Participants

Sixty-six inpatients with depression at the Psychiatric Department of Nanjing Brain Hospital participated in this study. Two or more physicians with a title of Attending or higher at the Psychiatric Department of Nanjing Brain Hospital conducted clinical diagnosis of MDD patients in accordance with the Diagnostic and Statistical Manual of Mental Disorders, 4th edition (DSM-IV). The Hamilton Rating Scale for Depression (HRSD) was used to assess the severity of depressive symptoms (27). The enrolled MDD patients met the diagnostic criteria of DSM-IV (28) and the index of the 10th edition of the International Classification of Diseases and Related Health Problems [ICD-10; (29)], and had no comorbidities other than DSM-IV 1-axis diseases. The total HRSD scores of 17 of the participants were ≥7 points. The exclusion criteria for MDD patients included (1) psychotropic medication in the past 2 weeks; (2) a history of alcohol/tobacco dependence or substance abuse; (3) received repeated transcranial magnetic stimulation or electroconvulsive physical therapy in the past 6 months; (4) contraindications to scanning by magnetic resonance imaging or magnetoencephalography; and (5) pregnancy. In this study, after the baseline visit, antidepressant medication was prescribed by each patient's clinician, as a real world study, we did not interfere with the patients' medication strategies. The actual medication status of the patient is shown in the **Table 2**.

This study was approved by the Medical Ethics Committee of Nanjing Medical University (2018-ky065-01) and all participants signed an informed consent form.

### MRI and MEG Data Acquisition and Analysis

Brain scans were conducted according to a previous study by our research group (30). Clean MEG data were frequency filtered into alpha [8–13 Hertz (Hz)] and beta (13–30 Hz) bands. The resting-state brain network related to depression was selected as the network of interest. The standard Montreal Neurological Institute (MNI) coordinates of each network brain area are shown in Table 1, including those of the DMN, CEN, and SN. We calculated the power of each brain network (alpha/beta-DMN, alpha/beta-CEN, alpha/beta-SN), the functional connections within the network (alpha/beta intra-DMN, alpha/beta intra-CEN, alpha/beta intra-SN), and the connections between one network and the other two networks (alpha/beta inter-DMN, alpha/beta inter-CEN, alpha/beta inter-SN) for each frequency wave.

**Table 1:** Network of interest and MNI coordinate.

**Network**	**Brain area**	**Abbreviation**	**MNI coordinate**		
			**X**	**Y**	**Z**
DMN	Posterior cingulate cortex	PCC	0	−50	20
	Anteromedial prefrontal cortex	amPFC	0	54	22
	Left angular gyrus	lAngGy	−49	−60	33
	Right angular gyrus	rAngGy	43	−63	31
CEN	L dorsolateral PFC	lDLPFC	−31	42	28
	R dorsolateral PFC	rDLPFC	35	44	28
	Dorsomedial PFC	dmPFC	0	25	41
	L superior parietal	lSupPar	−44	−47	44
	R superior parietal	rSupPar	44	−47	44
SN	Dorsal anterior cingulate	dACC	0	24	23
	L anterior insula	lAI	−41	−3	6
	R anterior insula	rAI	41	−3	6

### Statistical Analysis

Two-sample *t*-tests were used to compare differences between the response and non-response groups in the power and FC of each network in each band (alpha, 8–13 Hz; beta, 13–30 Hz). Correlations between power/FC and score reduction rate in HRSD-17 total scores and factors (anxiety/somatization, weight, cognitive, retardation, sleep), as well as the total score of baseline HRSD-17, were analyzed by Pearson correlation analysis (uncorrected). Receiver operating characteristic curves (ROCs) were constructed to determine thresholds for discriminating responder from non-responder patients.

## Results

### Patient Characteristics

The 66 depressed subjects were enrolled and completed a baseline MEG scan. However, 25 subjects left the program before it was completed, so only 41 subjects completed the week 4 MEG scan. *Response* was defined as a decline of >50% on the HRSD-17 following 4 weeks of antidepressant treatment (see Table 2).

**Table 2:** Participant demographics.

	**Non-response (***N*** = 11)**	**Response (***N*** = 30)**	**t/ X^**2**^/Z**	***P*** **-value**
Age	33 ± 9.8	31.0 ± 8.1	−0.65	0.519^a^
Education	13.5 ± 4.0	14 ± 2.7	0.535	0.596^a^
Gender (F/M)	5/6	11/19	0.261	0.723^b^
HRSD-17 (baseline)	26.1 ± 5.8	23.5 ± 5.0	−1.412	0.166^a^
This course (months)	5.0 ± 4.4	9.5 ± 11.0	−1.160	0.259^c^
Total course (months)	25.1 ± 41.8	41.0 ± 55.7	−1.001	0.331^c^
SSRIs	5	12	1.932	0.654^b^
SNRIs	4	14		
SSRIs+NaSSAs	2	2		
SNRIs+NaSSAs	0	2		

*SSRIs, selective serotonin reuptake inhibitors; SNRIs, selective serotonin and norepinephrine reuptake inhibitors; NaSSAs, noradrenergic and specific serotonergic antidepressants*.

a *Two sample T-test*.

b *Chi-square test*.

c *Rank sum test*.

### Differences in the Alpha/Beta Power/FC of Each Network Between Non-response and Response Patients

The difference at baseline between the response and non-response patients after treatment for 4 weeks was manifested in the beta power of the SN network (*t* = 2.264, *P* = 0.029, uncorrected). For further details, see the Supplementary Material.

### Correlation Between Network Interactions and Symptoms

The alpha power of DMN was correlated with the anxiety factor (*r* = 0.279, *P* = 0.023).

### Correlation Between the Alpha/Beta Power/FC of Each Network and Response

The beta FC of the inter-DMN was related to the relief of multiple symptoms and was negatively correlated with the reduction rates in HRSD-17 score (*r* = −0.38, *P* = 0.014) and anxiety (*r* = −0.49, *P* = 0.001). The beta wave FC of intra-CEN was correlated with the reduction in the retardation factor score (*r* = 0.33, *P* = 0.036). The beta FC of inter-SN was correlated with the anxiety reduction rate (*r* = −0.33, *P* = 0.036). And the beta FC of inter-CEN was correlated with the anxiety reduction rate (*r* = −0.31, *P* = 0.052).

### Value of the Alpha/Beta Power/FC of Each Network for Discriminating the Response

The beta power of the DMN and CEN also provided certain hints of response, as shown in the Table 3. The identification value increased after combining the three imaging indicators (AUC = 0.794, *P* = 0.004, sensitivity = 76.7%, specificity = 81.8%).

**Table 3:** ROC of each index.

**Image index**	**AUC**	***P*** **-value**	**Sensitivity (%)**	**Specificity (%)**
Beta-DMN	0.679	0.083	56.7	81.8
Beta-CEN	0.673	0.094	60	81.8
Beta-SN	0.742	0.019	56.7	90.1
Combined	0.794	0.004	76.7	81.8

## Discussion

In this study, mild depression patients (HRSD scores < 17) were also screened for inclusion. These patients were hospitalized and treated with antidepressants mainly because they wanted to recover as quickly as possible so that they can return normal life. Our findings suggested that interactions among the three core networks were associated with antidepressant efficacy. At baseline, compared to the non-response group, the response group exhibited a higher beta power value. After ROC analysis, it was found that the beta wave power of the three core networks at baseline helped predict the outcome of antidepressant treatment after 4 weeks. The average beta FC between the DMN network and the SN and CEN networks was negatively correlated with the total HRSD score reduction rate for depressive symptoms. The beta FC between the core networks was related to the relief of anxiety symptoms, while the internal CEN beta FC was positively correlated with the reduction in the retardation factor.

A recent study found that high beta-wave energy in the whole brain in the baseline resting state of depression suggests a better prognosis (21). The present study focused on three core networks, among which the SN network showed a difference at baseline; that is, the beta power of the SN network was higher in the response group. Emerging evidence suggests that the SN is an important regulating node in the interaction of neural network, and participates in the interaction between externally oriented attention and internally oriented self-related mental processes. Some scholars believe that the SN network plays a role in the regulation of DMN and CEN network switching, in turn, influences disease severity and core symptoms of major depression, such as anhedonia (31). Beta wave activity has been generally associated with top-down cognitive activity (17). Therefore, the high beta power of the SN network may indicate that the patient's SN can better actively coordinate and balance the functions of the CEN and DMN networks, so as to enable the patient to achieve better curative effects in the later period. Therefore, it is also helpful in predicting the treatment outcomes of depressed patients.

The beta FC of the beta-inter DMN was negatively correlated with the reduction rate in HRSD depressive symptoms. In general, the DMN network is dominated by alpha activity, so it may not be advantageous to connect the DMN to the beta waves of other networks. Notably, under normal circumstances, both during task performance and during rest, there is a clear segregation between DMN sites and task-positive sites in the CEN and SN (15). Widespread beta desynchronization in all three networks is observed during the performance of cognitively demanding tasks. However, in depressed patients, DMN activity is excessively coupled to SN activity (32). DMN dysfunction can cause patients to become trapped in a cycle of self-negative thinking and have no way to participate in coping with external stimuli. This is manifested most prominently in depressed patients for whom rumination leads to impaired cognitive resource allocation (32, 33). Therefore, the beta hypo-connection state of beta-inter DMN networks should indicate that the DMN causes little interference with the SN and CEN, and the brain is in a more reasonable working state.

The beta connection between each of the three core networks and the other two networks was negatively correlated with relief of anxiety symptoms, suggesting that independence of the three networks in the beta frequency band is beneficial to alleviating anxiety symptoms. Following the discussion in the previous paragraph, from the perspective of the DMN, its beta hypo-connections with the SN and CEN may indicate that these networks have little interference and that the brain is in a more reasonable working state; for example, with reduced rumination, or that the SN can better initiate network switching, leading to the disengagement of the DMN and engagement of the CEN.

The beta FC of the intra-CEN was positively correlated with a reduction in the retardation factor. A typical feature of MDD is the hypoconnectivity within the CEN network, resulting in a disability in emotional regulation and cognitive control of attention (4). Previous studies have shown that beta activity is negatively correlated with retardation symptoms (34) because the synchronized activity of beta waves helps regulate the psychomotor symptoms of patients with depression (35). Therefore, the increase in the FC of the intra-CEN may indicate that it can better exert attentional regulation and distribution, and cognitive and emotional control functions, thereby reducing the symptoms of retardation.

## Conclusions and Limitations

This study mainly explored the relationship between depression treatment efficacy and the interactions between the three core brain networks involved in depression, a topic that has received little research so far. The main finding is that the beta power of the SN and the beta FC of the DMN-SN/CEN are related to the overall efficacy of depression treatment. The beta wave connection between the three core networks is related to the relief of anxiety symptoms, while that of the intra-CEN is related to the relief of retardation symptoms. These results indicate the need for more in-depth exploration of the pathophysiological mechanisms of depression to understand the neural mechanisms of different depression sub-symptoms. The shortcomings of this study are that the sample size was low, so the clinical significance remains limited. Larger sample sizes could provide better verification. Secondly, we did not have a healthy control group for comparison.

## Data Availability Statement

The original contributions presented in the study are included in the article/Supplementary Material, further inquiries can be directed to the corresponding author/s.

## Ethics Statement

The studies involving human participants were reviewed and approved by the Medical Ethics Committee of Nanjing Medical University. The patients/participants provided their written informed consent to participate in this study.

## Author Contributions

QW and ST: conceptualization, data curation, formal analysis, and writing original draft. PZ and QC: conceptualization and writing original draft. QL: conceptualization and formal analysis. ZY: conceptualization. All authors contributed to the article and approved the submitted version.

## Funding

The authors are funded by the National Natural Science Foundation of China (81871066), the Jiangsu Provincial Key Research and Development Program (BE2018609 and BE2019675), the Jiangsu Provincial Medical Innovation Team of the Project of Invigorating Health Care Through Science, Technology and Education (CXTDC2016004), Key Project supported by the Medical Science and Technology Development Foundation, Jiangsu Commission of Health (K2019011), and the National Key R&D Plan (2018YFC1314601).

## Conflict of Interest

The authors declare that the research was conducted in the absence of any commercial or financial relationships that could be construed as a potential conflict of interest.

## Publisher's Note

All claims expressed in this article are solely those of the authors and do not necessarily represent those of their affiliated organizations, or those of the publisher, the editors and the reviewers. Any product that may be evaluated in this article, or claim that may be made by its manufacturer, is not guaranteed or endorsed by the publisher.
